# Nanoscale Porphyrin Metal-Organic Frameworks Deliver siRNA for Alleviating Early Pulmonary Fibrosis in Acute Lung Injury

**DOI:** 10.3389/fbioe.2022.939312

**Published:** 2022-07-18

**Authors:** Changmei Weng, Guanhua Li, Dongdong Zhang, Zhaoxia Duan, Kuijun Chen, Jieyuan Zhang, Tao Li, Jianmin Wang

**Affiliations:** ^1^ State Key Laboratory of Trauma, Burns and Combined Injury, Research Institute of Surgery, Daping Hospital, Third Military Medical University (Army Medical University), Chongqing, China; ^2^ Center for Joint Surgery, Southwest Hospital, Third Military Medical University (Army Medical University), Chongqing, China; ^3^ State Key Laboratory of Trauma, Burns and Combined Injury, Chongqing Engineering Research Center for Nanomedicine, Department of Preventive Medicine, Institute of Combined Injury, Third Military Medical University (Army Medical University), Chongqing, China

**Keywords:** porphyrin metal-organic framework, non-viral vector, siRNA, pulmonary fibrosis, endosomal escape

## Abstract

Acute lung injury (ALI) has high mortality and still lacks novel and efficient therapies. Zinc finger E-box binding homeobox 1 and 2 (ZEB1/2) are highly expressed in the early stage of ALI and are positively correlated with the progression of pulmonary fibrosis. Herein, we developed a nanoscale Zr(IV)-based porphyrin metal-organic (ZPM) framework to deliver small interfering ZEB1/2 (siZEB1/2) to alleviate early pulmonary fibrosis during ALI. This pH-responsive nano-ZPM system could effectively protect siRNAs during lung delivery until after internalization and rapidly trigger siRNA release under the mildly acidic environment of the endo/lysosome (pH 4.0–6.5) for transfection and gene silencing. Furthermore, the *in vivo* studies confirmed that this nano-ZPM system could anchor in inflamed lungs. Moreover, the ZEB1/2 silencing led to increased E-cadherin and decreased α-SMA levels. Overall, the nano-ZPM system was an excellent non-viral vector system to deliver siRNAs to alleviate early pulmonary fibrosis during ALI.

## Introduction

Acute lung injury (ALI) is associated with acute hypoxic respiratory insufficiency or respiratory failure, resulting from pulmonary injury during non-cardiogenic diseases such as severe infections, shocks, traumas, and burns (Fan et al., 2018; Bian et al., 2021). Additionally, ALI can develop into acute respiratory distress syndrome (ARDS) in more serious injuries (Matthay et al., 2019; Kaku et al., 2020). For example, the widespread of COVID-19 can cause ARDS, which is the primary factor leading to death. Although fluid management, glucocorticoids, and other therapeutic modalities have been applied to control ALI/ARDS, the clinical efficacy is still far from satisfactory (Matthay et al., 2017), leading to mortality as high as 30–50% (Beitler et al., 2022). Mechanical ventilation is the most important supportive treatment for patients with ALI/ARDS, but related complications such as ventilator-associated lung injury (VILI) and ventilator-associated pneumonia (VAP) also significantly affect the prognosis of ALI/ARDS patients (Aranda-Valderrama and Kaynar 2018; Zoulikha et al., 2022). Pharmaceutical drugs such as corticosteroids, lung surfactants, nitric oxide, and keratinocyte growth factors have been widely investigated for ALI/ARDS treatment. Unfortunately, none of them have been proved to effectively reduce the mortality of patients with ALI/ARDS in clinical trials (Qiao et al., 2021). In addition, many studies have demonstrated that the mesenchymal stem cell (MSC)-based therapy with anti-inflammatory, antifibrotic, anti-oxidative stress, and anti-apoptotic abilities is an alternative strategy to the treatment of ALI/ARDS (Fernandez-Francos et al., 2021). However, MSC treatment efficacy in ALI/ARDS is debated and controversial, so adequate mechanism research and functional test are needed before their clinical application. Consequently, the development of novel and efficient therapies for the prevention and treatment of ALI/ARDS is of great interest.

Pulmonary fibrosis can occur in the early stage of ALI, comprehending its crucial pathological process (Li et al., 2009; Burnham et al., 2014). The primary factors of ALI are the generation of reactive oxygen species (ROS) and the dysfunction of antioxidant systems. The epithelial-mesenchymal transition (EMT) in cells is also involved in those processes (Zhang et al., 2015; Kawami et al., 2022). The EMT is crucial for epithelial cells to acquire the mesenchymal phenotype and plays a critical role in the development of pulmonary fibrosis, mediated by different transcription factors (Zhou et al., 2016; Chen et al., 2017). The zinc finger E-box binding homeobox (ZEB) family is one of the important nuclear transcription factors, including Zinc-finger E-box binding homeobox 1/δ-crystallin E2-box factor 1 (ZEB1/δEF1) and Smad-interacting protein 1 (ZEB2/SIP1), important regulators of E-cadherin expression and the EMT (Chilosi et al., 2017). Silencing of both ZEB1 and ZEB2 can reverse the EMT and abolish the repression of epithelial splicing regulatory proteins (ESRP) induced by TGF-β in NMuMG cells (Horiguchi et al., 2012). Moreover, ZEB1/2 is positively correlated with the progression of early pulmonary fibrosis induced by lipopolysaccharide (LPS) (Cao et al., 2018). Therefore, ZEB1/2 can be considered novel target for the treatment of early-stage pulmonary fibrosis after ALI.

Small interference RNA (siRNA) technology has been widely used to explore gene function and gene therapy of malignant tumors, viral infections, autoimmune, and inflammatory diseases due to its high specificity (Kole et al., 2012; Weng et al., 2019; Hu et al., 2020). The clear anatomy, accessibility, and relatively low enzyme activity make the lung a good target for local siRNA therapy (Kandil and Merkel 2019; Zoulikha et al., 2022). For example, inhaled siRNAs can directly target and have long-term contact with pulmonary cells, which can be quickly effective and avoid first-pass elimination (Merckx et al., 2018; Zhu et al., 2022). However, large molecular weight (∼14 kDa), abundant negative charge, and poor stability of naked siRNAs limit their permeation across cell membranes (Wittrup and Lieberman 2015; Saeed et al., 2021). Thus, the development of new carriers for siRNA delivery into pulmonary cells is urgent. Nanoscale metal-organic frameworks (NMOFs) are a class of porous materials that have become promising candidates for drug encapsulation and delivery (Carrillo-Carrion 2020; Zhao et al., 2022). Recently, NMOFs have emerged as non-viral vector systems for delivering siRNA due to their high porosity, biodegradable structures, and controllable surface functionalities (He et al., 2014; Feng et al., 2020; Liu et al., 2022a). For example, Huang’s group developed a tumor-targeted, biomimetic manganese dioxide-shrouded metal-organic framework-based nanomedicine to deliver siRNA against the pyruvate kinase muscle isozyme M2 (siPKM2) to inhibit the reprogrammed glycolysis of triple-negative breast cancer cells (Huang et al., 2021). Herein, we reported the first use of NMOFs for siRNA delivery to treat ALI.

**SCHEME 1:**
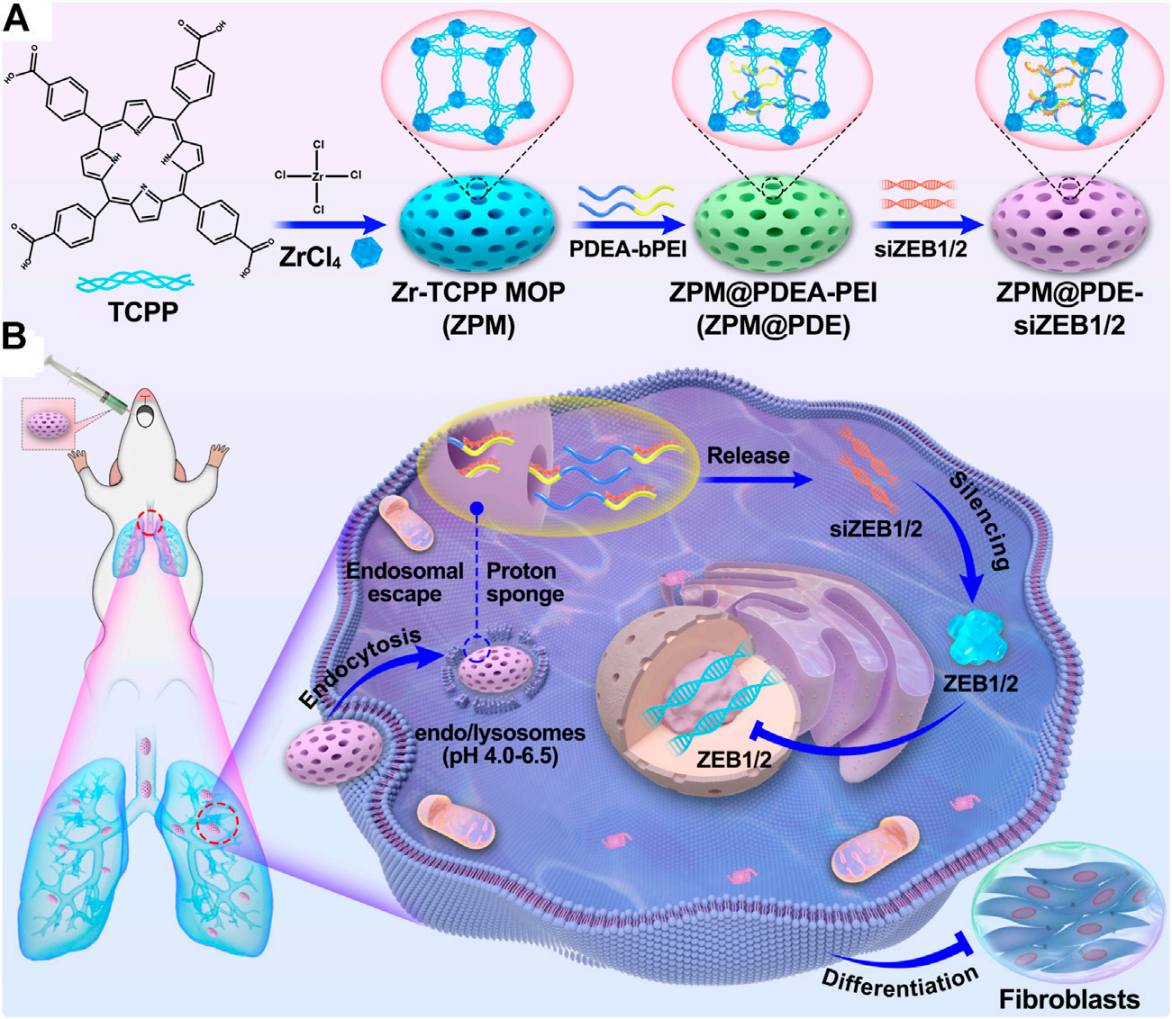
**(A)** The scheme for ZPM@PDE-siZEB1/2 preparation and **(B)** intratracheal therapeutic mechanism of ZPM@PDE-siZEB1/2 in ALI model mice.

In the present study, we designed a nanoscale Zr(IV)-based porphyrin metal-organic framework (ZPM) release system to deliver siZEB1/2 for alleviating early pulmonary fibrosis during ALI (Scheme 1). To promote the endosomal escape and cytosolic release of siZEB1/2, branched polyethyleneimine (bPEI) was conjugated to poly (2-diethylamino) ethyl methacrylate (PDEA) (abbreviate as PDE). Moreover, bPEI has a strong siRNA binding affinity and PDEA is ultra-pH-sensitive (pKa ≈6.4) and can aggregate into a hydrophobic core at pH 7.4 and undergo a charge transition to positive in acidic environments (Dai et al., 2018). Additionally, the ZPM-coated can protect the siRNA from enzymatic degradation in stable physiological pH 7.4 and decrease the cytotoxicity of bPEI. After ZPM@PDE-siZEB1/2 enters the cell through endocytosis, PDEA is quickly protonated in endo/lysosomes (pH 4.5–6.0), resulting in endosomal escape and cytosolic release of siRNA (Dai et al., 2017). Finally, the released siZEB1/2 can inhibit the expression of ZEB1/2, leading to EMT attenuation and alleviating early pulmonary fibrosis in LPS-induced ALI mice. This strategy might comprehend the basis for the smart design of a pulmonary siRNA delivery system to treat ALI/ARDS.

## Materials and Methods

### Materials

Branched polyethyleneimine (PEI, Mw 2 kDa), 2-(N,N-diethylamino)ethyl methacrylate (DEA), Cysteamine (95%), Azobisisobutyronitrile (AIBN), Succinic anhydride (SA), cis-Aconitic anhydride, dimethyl sulfoxide (DMSO), Triethylamine (TEA), pyridine, and acetone were purchased from J&K Scientific Ltd. (Beijing, China). ZrOCl2∙8H2O, Tetrakis (4-carboxyphenyl)porphyrin (TCPP), N,N-Dimethylformamide (DMF), N-ethyl-N-(3-(dimethylamino)-propyl) carbodiimide hydrochloride (EDC), and N-hydroxysuccinimide (NHS) were supplied by Aladdin Industrial Co., Ltd. (Shanghai, China). The siZEB1 (GTT​GTT​CTG​CCA​ACA​GTT​G) and siZEB2 (GGC​CGA​ATG​AGA​AAC​AAT​A) target both human and mice’s genes, and Cy5-labeled siZEB1/2 were designed and synthesized by RiboBio (Guangzhou, China). Lipofectamine 2000 was purchased from Invitrogen (Carlsbad, United States), and the LPS was obtained from Sigma-Aldrich (Missouri, United States). The Cell Counting Kit-8 (CCK-8) was bought from Boster Biological Technology (Wuhan, China). DAPI and total RNA Reagent Trizol were purchased from Life Technologies (New York, United States). The PrimeScript®RT Reagent Kit with gDNA Eraser was provided by TaKaRa (Beijing, China). Quantitative real-time qPCR kits (iTaq™ universal SYBR^®^ Green Supermix) were purchased from Bio-Rad (California, United States).

### Synthesis of PDEA-bPEI

**SCHEME 2:**
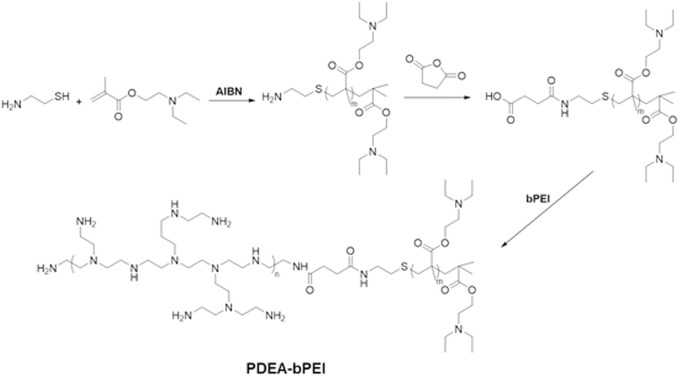
The synthetic scheme of PDEA-bPEI (PDE).

The PDEA-bPEI was synthesized as shown in Scheme 2. DEA (5.6 g, 30 mmol), Cysteamine (154.3 mg, 2 mmol), AIBN (16.4 mg, 0.1 mmol), and anhydrous THF (20 ml) were added into a 50 ml Schlenk tube under a nitrogen atmosphere. Then, the Schlenk tube was sealed, and freeze-pump-thaw degassing was performed to remove oxygen from the reaction tube. The mixture was immersed in an oil bath at 50°C under stirring for 4 h, followed by adding 8.0 ml of methanol to terminate the reaction. After cooling down, the crude product was repeatedly precipitated in cold n-hexane, filtered, and dried at 40 °C to generate oily PDEA (80% yield).

The linker SA-PDEA was synthesized as follows: in a three-port flask, PDEA (2.9 g, 1.0 mmol) and SA (150.1 mg, 1.5 mmol) were dissolved into DMSO/cis-Aconitic anhydride (1:5, 12 ml) under nitrogen atmosphere, then TEA (30 µL) and pyridine (30 µL) were added and continuously stirred for another 24 h at room temperature. The crude product was precipitated in cold n-hexane, filtered, and dried at 40°C under a vacuum to afford SA-PDEA. Next, SA-PDEA (2.4 g, 0.8 mmol), EDC (230.0 mg, 1.2 mmol) and NHS (138.1 mg, 1.2 mmol) were dissolved into DMSO (30 ml) under nitrogen atmosphere. After stirring at room temperature for 3 h, bPEI (0.8g, 0.4 mmol) was added and continuously stirred for another 24 h (overnight). First, the reaction solution was filtered, and the filtrate was added dropwise to water (150 ml), then purified by dialyzing (MWCO 3500) against Milli-Q water for another 3 days. The final product PDEA-bPEI (PDE) was obtained by freeze-drying.

### Preparation of ZPM@PDE-siZEB1/2

The zirconium porphyrin NMOFs (ZPM) was synthesized as follows: ZPM was synthesized using the solvothermal method with the morphology control of benzoic acid. ZrOCl_2_·8H_2_O (64.5 mg, 0.2 mmol), 25 mg of tetrakis (4-carboxyphenyl) porphyrin (26.8 mg, 0.04 mmol), and benzoic acid (0.73 g, 6 mmol) were dissolved into dry DMF solution (10 ml). Then, the mixture was vigorously stirred at 90°C for 5 h. After cooling down, the crude product was repeatedly washed with DMF and ethanol. The product was dispersed in ethanol for further use.

PDEA-bPEI was incorporated with ZPM as follows: PDEA-bPEI was dissolved in 10 ml of DMF (2 mg/ml), then 10 ml of the ZPM solution (2 mg/ml) was added and stirred for 24 h. ZPM@PDEA-bPEI (ZPM@PDE) was obtained by repeatedly washing with DMF and ethanol, then dried at 80°C in an oven for further use.

The ZPM@PDE-siZEB1/2 was prepared as follows: 1 mg of ZPM@PDE was dissolved in 1 ml RNase-free water. The siZEB1 and siZEB2 (each with 20 μM) were added and shaken for 12 h at 4°C in the dark. The ZPM@PDE-siZEB1/2 was collected by centrifugation and freeze-dried. The obtained product was stored at 4°C for further use. The Cy5-labeled siZEB1/2 of NMOF were prepared similarly. The binding ability of ZPM@PDE with siZEB1/2 was evaluated by agarose gel electrophoresis retardation assay (2% agarose gel, 80 V, 40 min).

### Material Characterization

The PDEA-bPEI was characterized by ^1^H NMR (400 MHz Agilent) and Fourier transform infrared (FT-IR) spectrophotometer (PerkinElmer S100, United States). Transmission electron microscopy (TEM, LIBRA 200 CS, Carl Zeiss Co., Germany), X-ray powder diffraction (XRD, PANalytical, Netherlands), thermal gravimetric analysis (TGA, Mettler Toledo, Switzerland), and N_2_ adsorption-desorption (ASAP 2460, Micromeritics, United States) were used to characterize the ZPM@PDE. The particle size and ζ-potential of ZPM@PDE were investigated by dynamic light scattering (DLS) (Nano ZS90 Zeta sizer, Malvern Instruments Co., Ltd., United Kingdom).

### Release of siRNAs

To quantify the release rate of siZBE1/2, ZPM@PDE-siZBE1/2 was cultured in PBS at 37°C and pH 5.0 or pH 7.4. At predetermined time points, samples from each group were centrifuged, then the concentration of siZBE1/2 in the supernatant was measured using a spectrophotometer. All release experiments were conducted in triplicates.

### Cytotoxicity *In Vitro*

The cytotoxicity of the ZPM@PDE-siZBE1/2 was determined by CCK-8 with the A549 cell line. Briefly, A549 cells were cultured in 96-well plates with an initial density of 8 × 10^3^ cells per well and cultured for 24 h, then incubated with 10 μL of ZPM@PDE-siZBE1/2 and PDE-siZEB1/2 at different concentrations (0, 20, 50, 100, 200, and 500 μg/ml) in complete medium for 24 h. Finally, 10 μL of CCK-8 solution was added per well and incubated for 2 h. The absorbance was measured on a microplate reader (Varioskan LUX, Thermo Scientific, MA, United States) at 450 nm.

### Cellular Uptake and Gene Silencing Efficiency *In Vitro*

First, A549 cells were inoculated in 6-well plates (2 × 10^5^ cells per well) or 12-well plates (1 × 10^5^ cells per well) and cultured for 24 h. Before the NMOF was added, the growth medium was replaced with fresh DMEM (pH 7.4 or 5.0). Then, ZPM@PDE-Cy5-siZEB1/2 (20 μg/ml) was added to each well and co-cultured for another 12 h. Cells were gently washed with PBS to remove redundant ZPM@PDE-Cy5-siZEB1/2. Cells seeded in 6-well plates were harvested for total RNA extraction. Cells seeded in 12-well plates were fixed with 4% paraformaldehyde for 15 min at room temperature. The nuclei were co-stained with 4′,6-diamidino-2-phenylindole (DAPI) and imaged using a fluorescence microscope (Olympus VS.200 ASW and Olympus BX48, Japan). The treatments were Lipofectamine 2000-Cy5-siZEB1/2 (Lip 2000-Cy5-siZEB1/2), naked Cy5-siZEB1/2, and fresh DMEM (control).

### Animal Experiments

All animal experiments were performed following the regulations and guidelines of the Ethics Committee for Animal Experiments and were approved by the Ethics Committee of the Third Military Medical University. C57BL/6 mice (18–22 g, 6 weeks) were obtained from the animal center of the Daping Hospital (Chongqing, China). Mice were maintained in specific pathogen-free (SPF) conditions with free access to water and laboratory rodent food. The LPS-induced ALI mice model was established as follows: after being anesthetized with 0.1% pentobarbital (30 mg/kg of body weight), mice were fixed on cardboard with a 60° slope in the supine position and an intratracheal quantitative drug delivery device (YuYan instruments, Shanghai, China) was applied. Mice were treated with LPS (5 mg/kg), naked siZEB1/2, and saline (control). After LPS instillation, mice tracheal inhaled ZPM@PDE-siZEB1/2 or ZPM@PDE-Cy5-siZEB1/2 at 0.5 h. Treated mice were observed for 3 days, then sacrificed. Lung tissues were obtained for imaging, histopathologic analysis, and total RNA and protein extraction.

### *Ex Vivo* Lung Imaging Analysis

The lungs of C57BL/6 mice that tracheal inhaled LPS, then ZPM@PDE-Cy5-siZEB1/2 were collected and rinsed with PBS. Mice were treated with the same volume of saline as the control. Fluorescence images of the lungs were acquired using an *in vivo* imaging system (IVIS Spectrum, Perkin Elmer, United States).

### Histological Examination

To observe the pathological changes in lung tissues, including inflammatory cell infiltration, pulmonary edema, hemorrhage, and pulmonary structural changes, lung tissue samples were fixed with 4% paraformaldehyde, embedded in paraffin, and sliced (5 μm thick). After representative organ sections were deparaffinized, hydrated, and stained with hematoxylin-eosin (H&E) or Masson’s trichrome staining, they were observed under a microscope (Olympus VS.200 ASW and Olympus BX48, Japan). The semi-quantitative scoring on lung injury degree was determined using the Smith score (Smith et al., 1997): in each field, inflammation, hemorrhage, and edema, in both alveolar and interstitium, were graded on a 0–4 point scale (0: no injury, absent or appears normal, 0%; 1: light, < 25%; 2: moderate, 25–50%; 3: strong, 50–75%; 4: intense, > 75%). The pulmonary fibrotic grade of each field was assayed via the Aschoff score criteria as previously described (Chen et al., 2013). Five sections pictured from each lung were analyzed, and three independent observers marked the score.

### Isolation of RNA and Quantitative Real-Time qPCR

Total RNA was extracted from A549 cells and lung tissues with Trizol, and reverse transcribed with PrimeScript®RT Reagent Kit according to the manufacturer’s instructions. Then, cDNA was subjected to 10 μL real-time qPCRs and implemented in CFX96TM Real-Time System using SYBR Green Supermix (Bio-Rad Company, United States). GAPDH and β-actin were used as reference genes. Data are presented as the fold change relative to the control using the 2^−ΔΔCt^ method and the expression levels for the target gene were calculated by the comparative threshold cycle (CT) method. Primer sequences are shown in Supplementary Table S1.

### Total Protein Extract for Western Blotting Analysis

Total protein was extracted from A549 cells and lung tissues using a total protein extraction kit. Protein samples were separated by 8% sodium dodecyl sulfate (SDS)-polyacrylamide gel and transferred to PVDF membrane by electrophoresis, then blocked with 5% non-fat milk at room temperature for 2 h. After membranes were incubated overnight with ZBE1, ZBE2, and β-actin antibodies at the appropriate dilution at 4°C, horseradish peroxidase (HRP)-linked secondary antibody was used for detection by Immobion™ Western Chemiluminescent (Millipore, United States). The band’s density was quantified using Image-Pro Plus software (Version 6.0; Media Cybernetics, Silver Springs, MD, United States). β-actin was used as the internal control to normalize protein levels.

### Immunofluorescence Staining and Immunohistochemistry Assay

For the immunofluorescence assay, the specific operation before incubation of the second antibody was the same as the immunohistochemistry staining. After incubating with E-cadherin and α-SMA primary antibodies under 4°C overnight, slides were washed with PBS buffer, then incubated with Cy3 or FITC fluorescent secondary antibody. Finally, nuclei were co-stained with DAPI. E-cadherin and α-SMA expression levels were observed under a fluorescence microscope.

Immunohistochemistry staining for detection of E-cadherin and α-SMA was performed as follows. Briefly, paraffin-embedded slices were cleared and antigen repair was performed. Then, endogenous peroxidase activity was quenched with an endogenous peroxidase blocker, and samples were incubated with E-cadherin or α-SMA primary antibodies at 4°C overnight. The target protein was recognized using horseradish peroxidase-conjugated and DAB chromogenic solution, then images were observed under a microscope.

### Statistical Analysis

Data are presented as means ± SDs (standard deviations). Statistical analyses were conducted with SPSS 23 (IBM.United States) using one-way ANOVA or Student’s *t*-test. A *p* < 0.05 was considered statistically significant.

## Results and Discussion

### Fabrication and Characterization of ZPM@PDE

The PDEA-bPEI (PDE) was successfully synthesized as confirmed by ^1^H NMR and FT-IR (Supplementary Text and Supplementary Figures S1, S2). In the ^1^H-NMR spectrum (Supplementary Figure S1), the bPEI peak appeared at 2.46–2.81 ppm. In the PDE, the double peak at 2.40–2.81 ppm was attributed to the presence of methylene protons (-N-CH_2_CH_2_-N-) in the bPEI. Signals at δ = 0.72–1.21 ppm and *δ* = 3.99 ppm corresponded to the -CH_3_ and (-O-CH_2_-) protons of PDEA, respectively. In the FT-IR spectrum (Supplementary Figure S2), the strong absorption peak at 3,438 cm^−1^ was attributed to the–NH_2_ on bPEI. The strong absorption peaks at 2,970 cm^−1^ and 1,150 cm^−1^ were attributed to the characteristic absorption peak of–CH_3_ and O-C bonds stretching vibration on PDEA, respectively. These results confirmed that the PDE was successfully synthesized.

**FIGURE 1:**
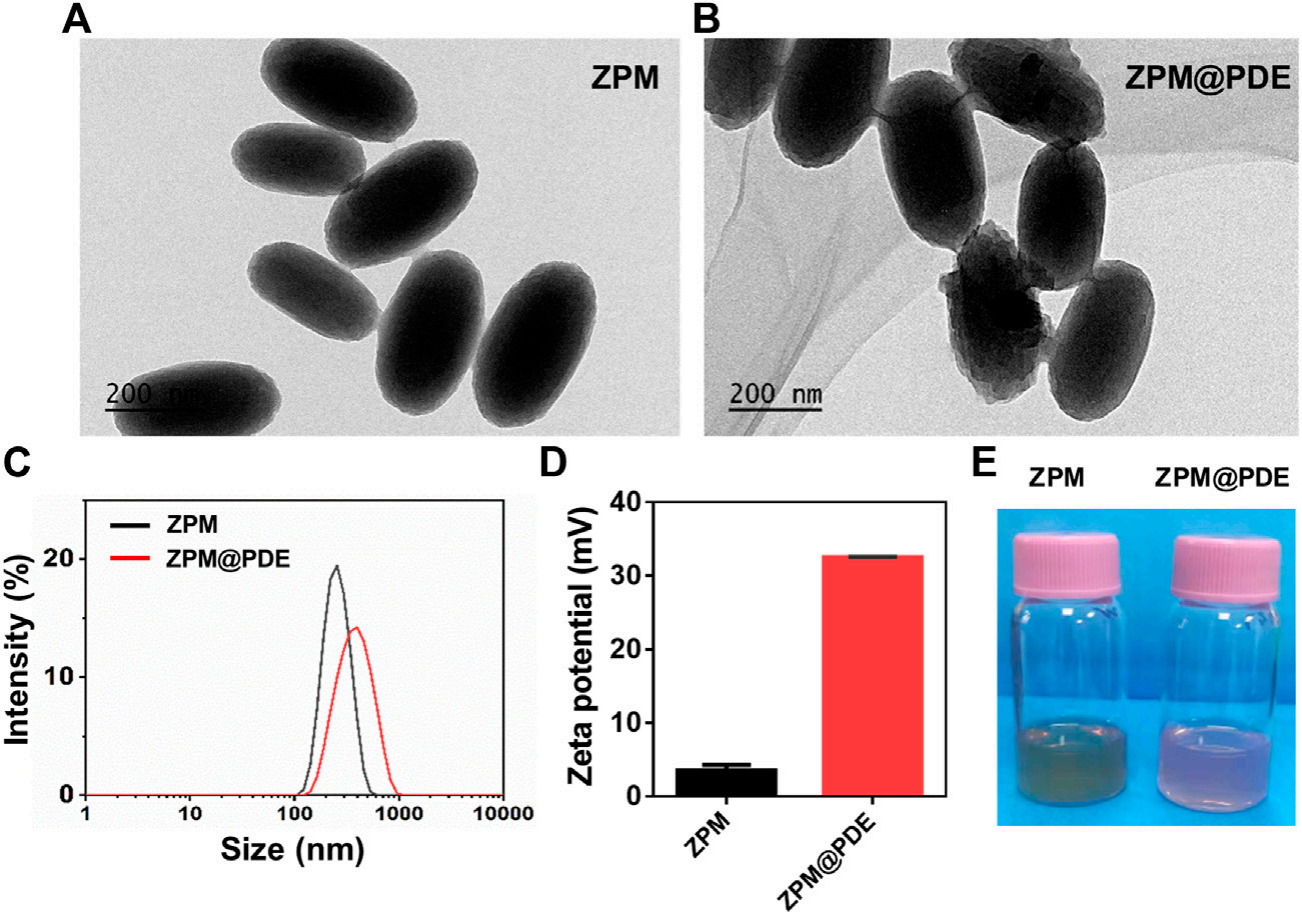
**(A,B)** TEM of TZM and TZM@PDE, **(C)** Hydrodynamic size distribution, **(D)** Zeta potential, and **(E)** Photographs of TZM and TZM@PDE in aqueous solutions.

Further, the ZPM was successfully synthesized by solvothermal methods. The ZPM@PDE was obtained via ZPM adsorption with PDE by blending for 24 h. Based on the SEM, the morphology of ZPM and ZPM@PDE was of uniform monodispersed nanoshuttles (Figures 1A,B). The hydrodynamic size distribution increased from 240.6 to 330.4 nm and the zeta potential increased from 3.5 ± 0.4 to 32.5 ± 0.3 mV with PDE incorporation according to the dynamic light scattering (Figures 1C,D). Furthermore, the colors changed from green to pink (Figure 1E). These results showed that PDE was successfully wrapped in ZPM.

**FIGURE 2:**
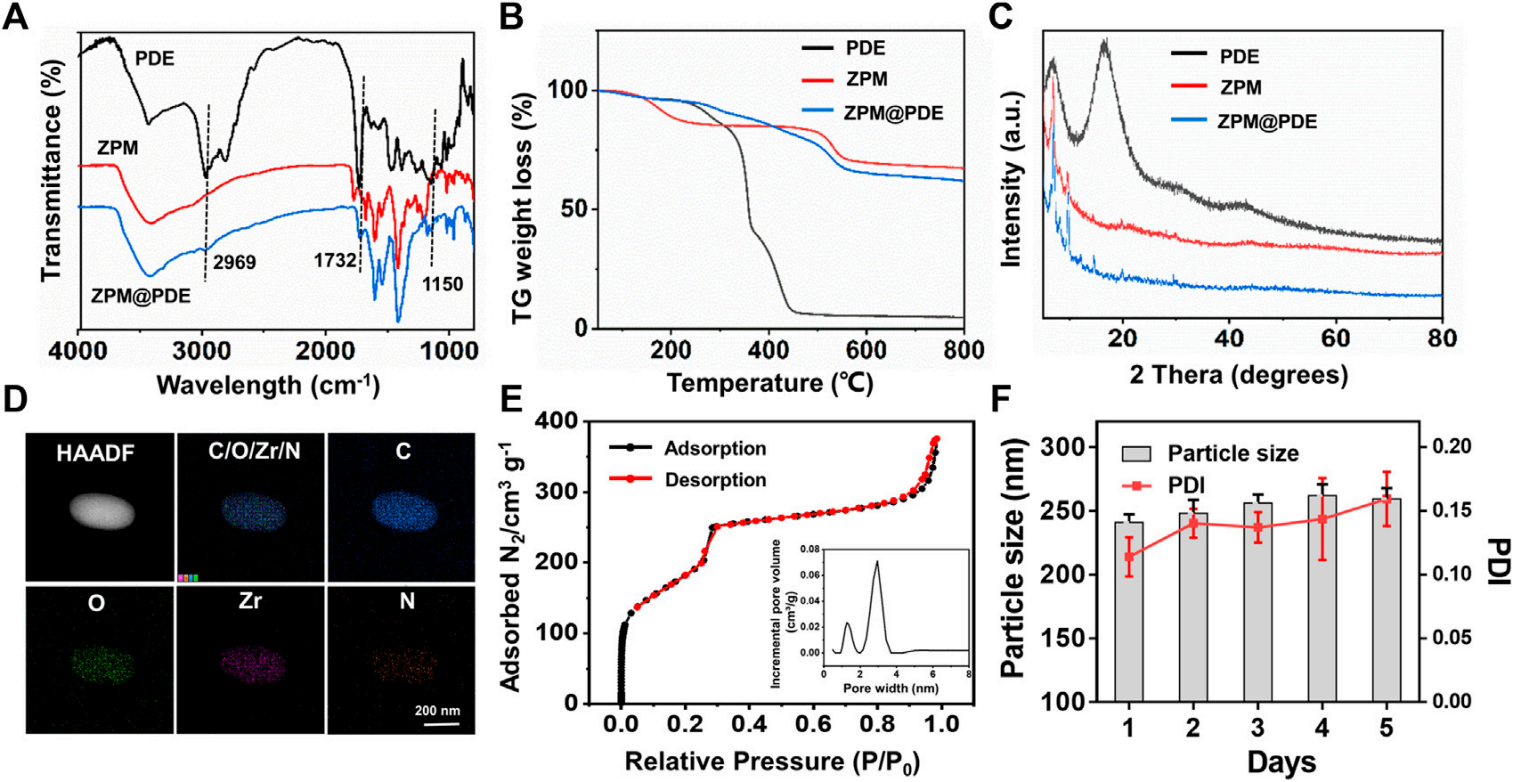
**(A)** Fourier transform infrared (FT-IR) spectra, **(B)** TG weight loss profiles, **(C)** XRD patterns of PDE, ZPM, and ZPM@PDE, **(D)** HR-TEM and elemental mapping images of ZPM@PDE, **(E)** N_2_ adsorption-desorption isotherms and DFT pore size distribution of ZPM, and **(F)** Hydrodynamic size distribution and PDI of ZPM in H_2_O for 5 days.

Then, FT-IR, powder X-ray diffraction (XRD), thermogravimetric analyzer (TGA), HR-TEM, and N_2_ adsorption-desorption were used to validate the successful incorporation of PDE to ZPM. The FT-IR spectra of the ZPM@PDE had new characteristic peaks at 2,969, 1732, and 1,150 cm^−1^ assigned to the vibration of -NH2 in the PDE, which indicated the presence of PDE in ZPM@PDE (Figure 2A). Additionally, the TGA results showed that ZPM@PDE had lower heat flow intensity compared to ZPM due to the poor thermal stability of PDE (Figure 2B). Furthermore, the powder X-ray diffraction (XRD) pattern of ZPM and ZPM@PDE were consistent, which demonstrated that ZPM@PDE maintained the same crystal structure as ZPM during the incorporation process (Figure 2C). In addition, the HR-TEM images with elemental mapping showed that N was uniformly distributed in the particles (Figure 2D). Overall, these results further demonstrated that PDE was successfully wrapped in ZPM. In addition, the surface area and average pore diameter of ZPM were 775.8 m^2^/g and 2.6 nm, respectively. (Figure 2E). Consistent with previous literature, our results exhibited the similar structure and pore size distribution with the acid-resistant mesoporous MOF (PCN-222) (Wang et al., 2019). DLS verified that ZPM had excellent colloidal stability in H_2_O for at least 5 days (Figure 2F) verifying ZPM was suitable as a delivery carrier.

### siRNA Loading Affinity of ZPM@PDE and Release Profile

**FIGURE 3:**
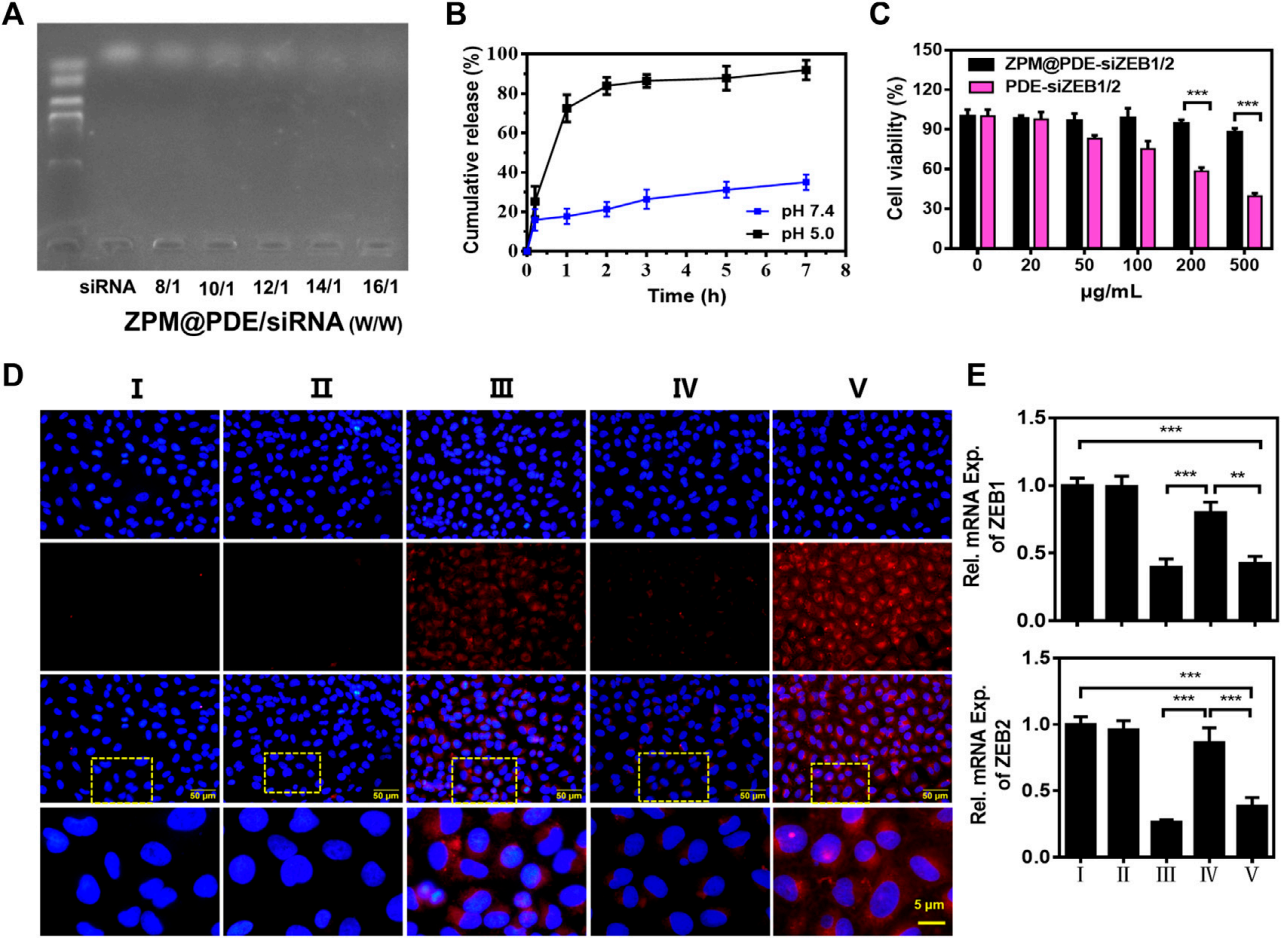
**(A)** Gel retardation assay of ZPM@PDE/siZEB1/2 with different mass ratio, **(B)** siZEB1/2 release from ZPM@PDE-siZEB1/2 at pH 5.0 or pH 7.4 over time, **(C)** Cytotoxicity of PDE@siZEB1/2 and ZPM@PDE-siZEB1/2, **(D)** Uptake of ZPM@PDE-Cy5-siZEB1/2 (red) *in vitro*. Nuclei stained with DAPI nuclear dye (blue), and **(E)** Silencing efficacy of ZPM@PDE-siZEB1/2 *in vitro*. (*n* = 3, *p < 0.05, **p < 0.01, ***p < 0.001). For each group: Ⅰ, Control; Ⅱ, Naked CY5-siZEB1/2; Ⅲ, Lip 2000-CY5-siZEB1/2; Ⅳ, ZPM@PDE-CY5-siZEB1/2 (pH 5.0); Ⅴ, ZPM@PDE-CY5-siZEB1/2 (pH 7.4).

The ZPM@PDE-siZBE1/2 was prepared by incubating ZPM@PDE with siZEB1/2. The siRNA binding ability of ZPM@PDE was detected by gel retardation assay. The bright band gradually disappeared with increasing ZPM@PDE/siZEB1/2 mass ratios (Figure 3A). When the mass ratio reached 14/1, the bright band completely disappeared, demonstrating that the siZBE1/2 was tightly bonded. The release profile of siZBE1/2 from ZPM@PDE/siZEB1/2 was measured in PBS at different pH values for 7 h (Figure 3B). Under physiological conditions (pH 7.4), the release of siZBE1/2 was less than 40% after 7 h. On the other hand, under the endo/lysosome mimic condition (pH 5.0), the release of siZEB1/2 reached more than 80% within 2 h, indicating that ZPM@PDE/siZEB1/2 had a pH-responsive feature. This phenomenon also indicated that PDEA segments can transform from hydrophobic to hydrophilic when the environmental pH is reduced (pH < 6.4) (Dai et al., 2017; Dai et al., 2018). Therefore, the NMOF could effectively protect the siRNA during lung delivery until after internalization, and rapidly trigger siRNA release under the mildly acidic environment of the endo/lysosome (pH 4.0–6.5), thereby allowing siRNA transfection and gene silencing (Zhuang et al., 2014; Zhuang et al., 2020).

### Cytotoxicity, Cellular Uptake, and Gene Silence Ability of ZPM@PDE-siZEB1/2

Furthermore, we investigated the cytotoxicity of ZPM@PDE-siZEB1/2 on A549 cells using the CCK-8 assay (Figure 3C). The cell viability in ZPM@PDE-siZEB1/2-treated cells was higher than in PDE-siZEB1/2-treated cells with the same concentration. This result verified that the ZPM outer layer improved the biocompatibility of the NMOF. Moreover, the ZPM@PDE-siZEB1/2 presented excellent biocompatibility with cell viability >87.9%, even when the concentration of ZPM@PDE-siZEB1/2 was up to 500 μg/ml. Similarly, the nanoscale ZMP exhibited enhanced cellular uptake and excellent biocompatibility for intracellular delivery of proteins in the research of Wang et al. (2019).

Next, the fluorophore Cy5-labeled siZEB1/2 was used to evaluate the cellular delivery of ZPM@PDE-siZEB1/2 at different pH levels (Figure 3D). ZPM@PDE-CY5-siZEB1/2-treated A549 cells exhibited higher cellular red fluorescence of Cy5-labeled siZEB1/2 at pH 7.4 than pH 5.0. When ZPM@PDE-siZEB1/2 are in the mild acidity condition (pH 5.0), PDEA segments can be quickly protonated resulting in siRNA release out of the cells, losing the ability to pass through cell membranes. The fluorescence signal of ZPM@PDE-CY5-siZEB1/2-treated A549 cells was similar to Lip2000-siZEB1/2-treated cells, which might ascribe indicate that the siRNA delivery system accelerated the endocytosis by interacting with anionic proteoglycans on the cell surface (Subhan and Torchilin 2020).

To investigate the gene silencing efficiency of siZEB1/2 deliver by ZPM@PDE-siZEB1/2, A549 cells were transfected with ZPM@PDE-siZEB1/2 at different pH levels and analyzed by real-time qPCR (Figure 3E). The ZPM@PDE-siZEB1/2 showed significantly lower ZEB1 and ZEB2 gene levels at pH 7.4 than pH 5.0. These data supported that ZPM@PDE-siZEB1/2 could efficiently deliver siZEB1/2 to cells and mediate gene silence, especially under physiological conditions.

### ZPM@PDE-siZEB1/2 Delivers siZEB1/2 to the Lungs

**FIGURE 4:**
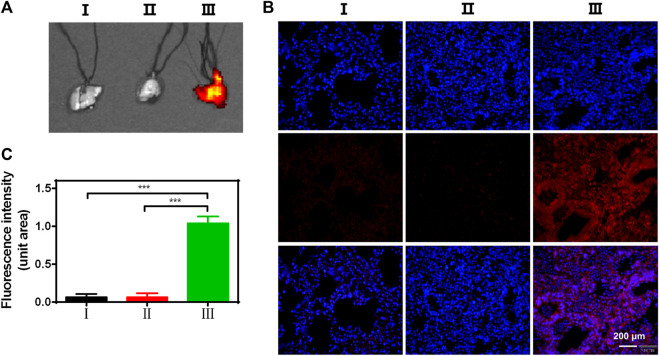
**(A)** Fluorescent images of lungs, **(B)** Uptake of CY5-siZEB1/2 (red) in mice, and **(C)** The relative fluorescence intensity of CY5. Nuclei stained with DAPI nuclear dye (blue). For each group: Ⅰ, Control; Ⅱ, LPS + Naked CY5-siZEB1/2; Ⅲ, LPS + ZPM@PDE-CY5-siZEB1/2.

In ALI/ARDS treatment, siRNAs have been previously confirmed as an effective nucleic acid-based therapy. However, siRNAs can be easily degraded by enzymes during transport progress (Jagrosse et al., 2019; Ge et al., 2020). Therefore, the delivery ability of siRNAs in the lungs is an important factor to evaluate an optimal siRNA delivery vector. To explore the lung delivery ability, ZPM@PDE-CY5-siZEB1/2 was inhaled by LPS-induced ALI mice and normal mice using the intratracheal quantitative drug delivery method. The lungs collected were imaged with an *in vivo* imaging system (Figure 4A). The LPS + ZPM@PDE-Cy5-siZEB1/2 group presented much stronger fluorescence in the lung compared to the LPS + Naked Cy5-siZEB1/2-treated group. The fluorescence of Cy5 was vividly observed in the LPS + ZPM@PDE-Cy5-siZEB1/2 group, indicating that siZEB1/2 was successfully delivered into the lungs. Meanwhile, the fluorophore Cy5-labeled siZEB1/2 was also identified in lung tissue sections (Figures 4B,C). Moreover, the tissue sections presented uniformly distributed high fluorescence intensity, demonstrating that the siZEB1/2 were also delivered to pulmonary local tissues including the depth part.

### ZPM@PDE-siZEB1/2 Alleviates Early Pulmonary Fibrosis in ALI Mice

**FIGURE 5:**
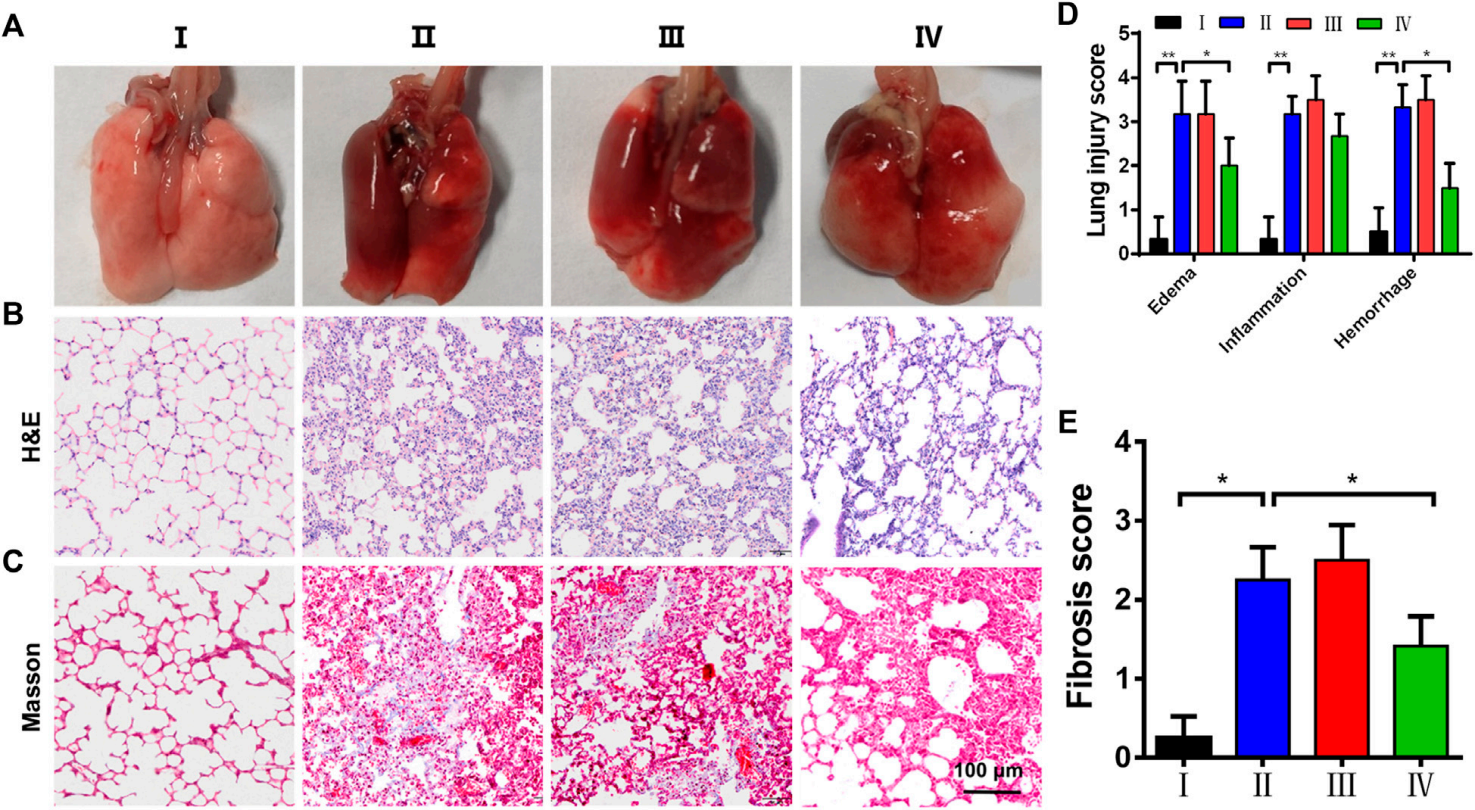
The histopathological change of lung tissues. **(A)** Macroscopic appearance of lungs, **(B,C)** H&E and Masson’s trichrome staining of lungs; **(D)** pulmonary injury score (Smith score); and **(E)** pulmonary fibrosis score (Aschoff score) from mice treated with saline (control), LPS, LPS + Naked siZEB1/2 and LPS + ZPM@PDE-siZEB1/2. (*p < 0.05, **p < 0.01, ***p < 0.001). For each group:Ⅰ, Control; Ⅱ, LPS; Ⅲ, LPS + Naked siZEB1/2; Ⅳ, LPS + ZPM@PDE-siZEB1/2.

Based on the siZEB1/2 could be effectively delivered to pulmonary local tissues, an ALI model was developed by intratracheal administration of LPS to evaluate the therapeutic function *in vivo*. The macroscopic observation of the lungs is shown in Figure 5A. In the control group, the lungs showed pink, soft, and clear texture, while the LPS and LPS + Naked siZEB1/2 groups showed dark red with obvious damage. While treated with ZPM@PDE-siZEB1/2, the symptoms were significantly alleviated. The histopathological change of lung tissues was examined using H&E and Masson staining (Figures 5B,C). H&E staining results showed that alveolar walls thickening, alveolar collapse, and inflammatory cell infiltration were obviously observed in LPS and LPS + Naked siZEB1/2 groups. In contrast, these pathological changes were significantly alleviated in LPS + ZPM@PDE-siZEB1/2 group. Consistently, the lung injury scores including edema, inflammation, and hemorrhage of LPS + ZPM@PDE-siZEB1/2 group were significantly lower than that of LPS and LPS + Naked siZEB1/2 groups, but there was no significant difference between LPS + ZPM@PDE-siZEB1/2 group and control group (Figure 5D). Moreover, Masson staining results revealed that a large number of collagen fibers appeared around the vessels and bronchi in LPS group, which indicated the typical pulmonary fibrosis formation. Treatment with ZPM@PDE-siZEB1/2 effectively rescued these changes. Similarly, the fibrosis score of LPS + ZPM@PDE-siZEB1/2 group was also lower than that of LPS group (Figure 5E). Collectively, These results revealed that simultaneous silencing of ZEB1 and ZEB2 in local lung tissue could alleviate LPS-induced early pulmonary fibrosis. For delivering siRNA to impede ZEB1 and ZEB2, PEI has been widely used to deliver siRNA in many fields due to its high gene condensation ability, quick endosome escape activity, and high transfection efficiency (Nimesh 2012; Sung and Kim 2020; Zoulikha et al., 2022). However, the positive charge of PEI with molecular weight-dependent cytotoxicity could led to apoptosis and necrosis of cells limiting its final use in the clinic (Nimesh et al., 2011; Cavallaro et al., 2017). RNA transfection reagent jetPEI could deliver paxillin siRNA AG975 by intratracheal administration to attenuate LPS-induced paxillin tyrosine phosphorylation and lung injury in mice (Fu et al., 2015). To improve the shortcomings of PEI in the use of ALI, we developed nanoscale MOFs with high porosity, high specific surface area, and good biocompatibility to cover PEI. MOFs have been proved to be an effective delivery carrier in the lungs (Zuo et al., 2020; Liu et al., 2022b).

**FIGURE 6:**
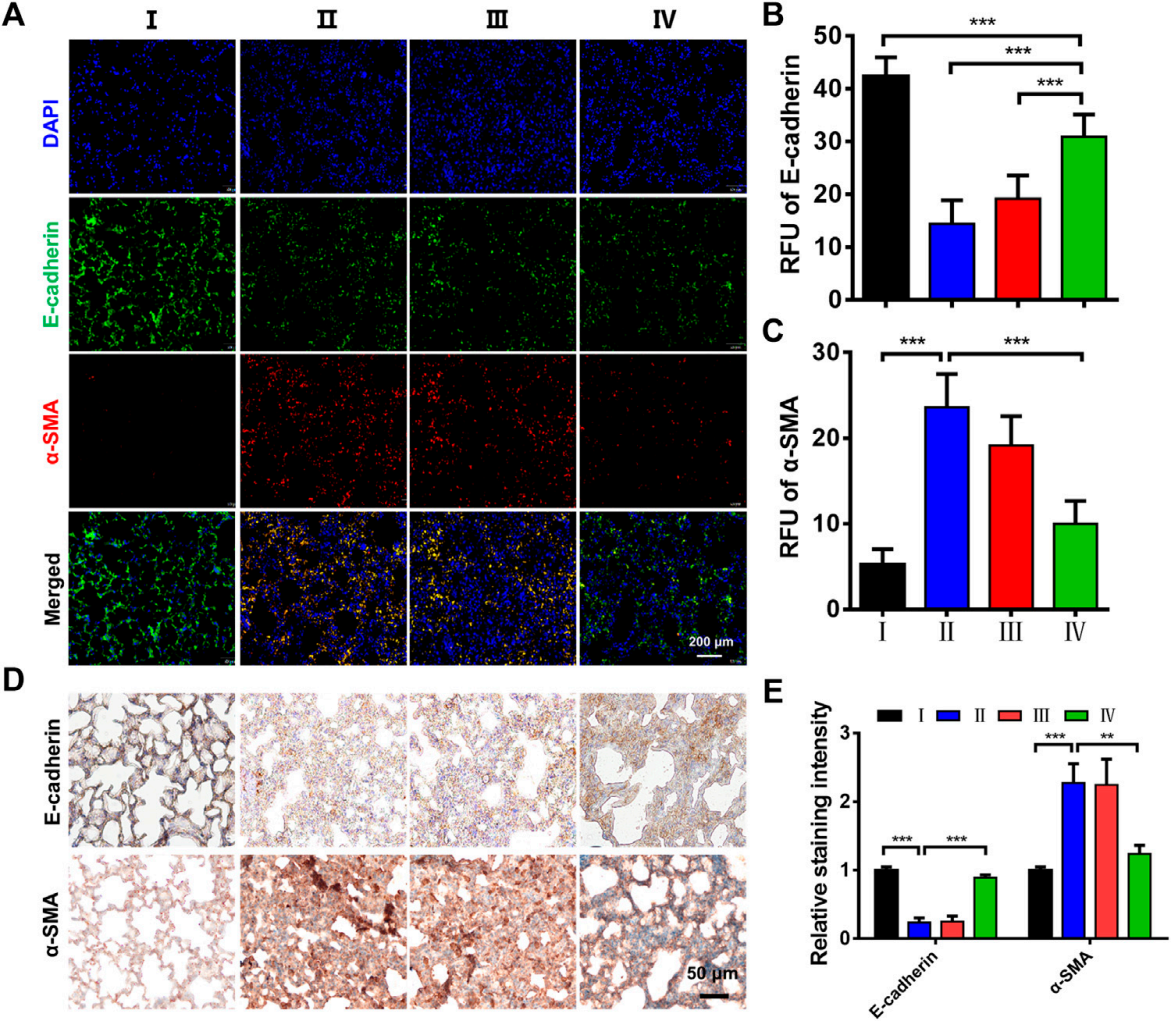
Effects of ZPM@PDE-siZEB1/2 on the expression of E-cadherin and α-SMA in mice. **(A)** Immunofluorescence assays expression of E-cadherin (green) and α-SMA protein (red), nuclei stained with DAPI nuclear dye (blue), **(B,C)** The relative fluorescence unit (RFU) of E-cadherin and α-SMA, **(D)** Immunohistochemical analysis expression, and **(E)** The relative staining intensity of E-cadherin and α-SMA. (*p < 0.05, **p < 0.01, ***p < 0.001). For each group:Ⅰ, Control; Ⅱ, LPS; Ⅲ, LPS + Naked siZEB1/2; Ⅳ, LPS + ZPM@PDE-siZEB1/2.

Furthermore, the EMT plays a crucial role in the development of early pulmonary fibrosis, accompanied by the change of molecular biomarkers (Tellez et al., 2011; Nagaraja and Nagarajan 2018). The epithelial marker E-cadherin and mesenchymal marker α-SMA are well-accepted markers to evaluate the EMT. Herein, the immunofluorescence images showed that LPS treatment significantly decreased the levels of E-cadherin and increased the levels of α-SMA (Figure 6A). Meanwhile, the ZPM@PDE-siZEB1/2 treatment resulted in an E-cadherin increase and α-SMA decrease. The relative fluorescence unit (RFU) statistical analysis also showed an increase in E-cadherin and a decrease in α-SMA expression in the LPS + ZPM@PDE-siZEB1/2 group (Figures 6B,C). The immunohistochemical staining presented similar results (Figures 6D,E). Overall, the abovementioned results together demonstrated that the ZPM@PDE-siZEB1/2 is effective and safe in treating the ALI by delaying EMT progress in the development of early pulmonary fibrosis.

### Gene Silencing Ability of ZPM@PDE-siZEB1/2 in Lungs

**FIGURE 7:**
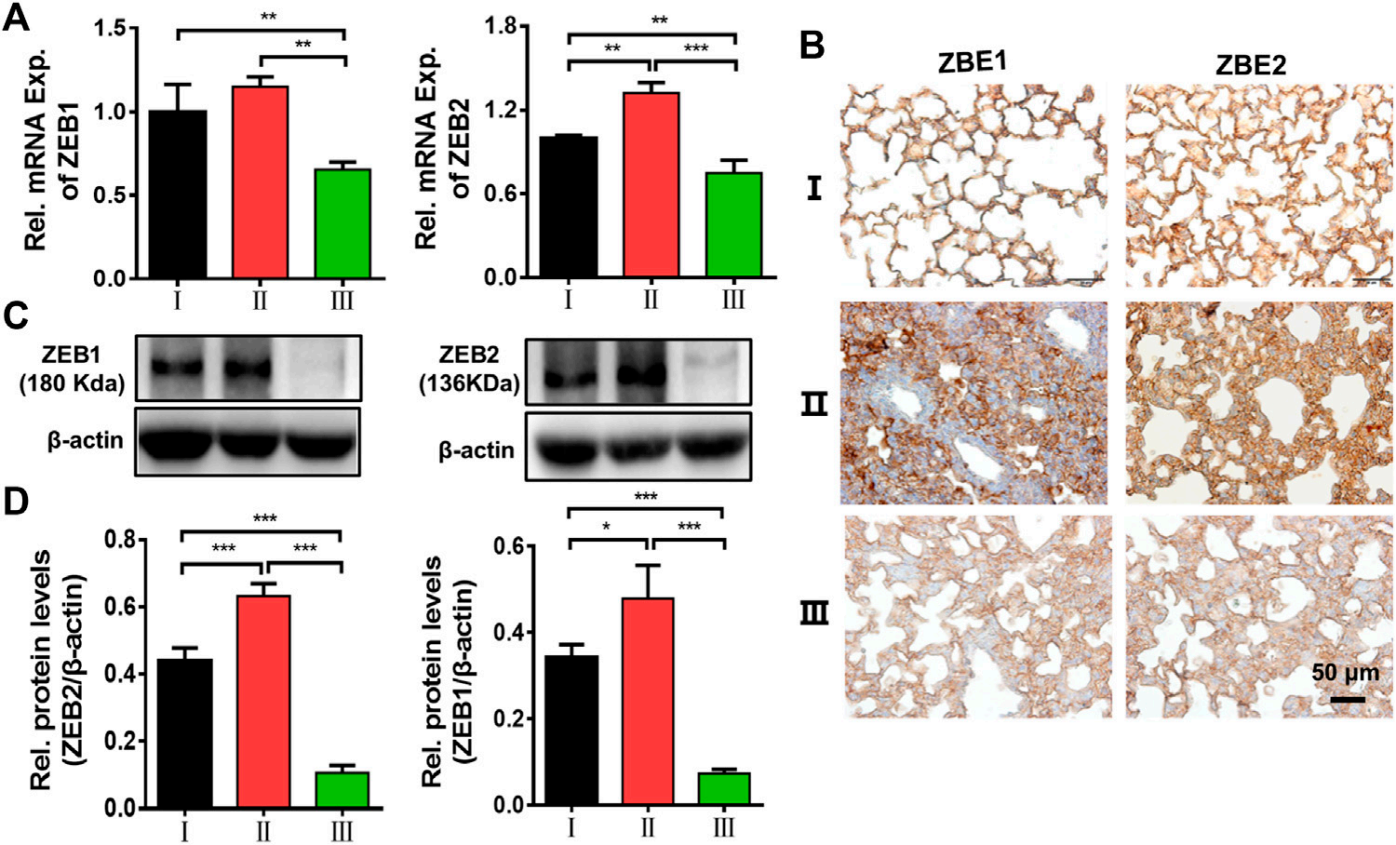
Effects of ZEB1/2 silencing ability *in vivo*. **(A)** Immunohistochemical analysis expression of ZEB1/2 in mice lungs, **(B)** expression of ZEB1/2 were detected by quantitative RT-PCR, and **(C,D)** western blot analysis. (*p < 0.05, **p < 0.01, ***p < 0.001). For each group:Ⅰ, Control; Ⅱ, LPS + Naked siZEB1/2; Ⅲ, LPS + ZPM@PDE-siZEB1/2.

*In vitro* and *in vivo* results have shown that our delivery system as a type of siRNA vector has a good anti-pulmonary fibrosis feature in ALI. Finally, the possible mechanism of action was examined. Real-time qPCR, immunohistochemical staining, and Western blotting for ZEB1/2 were performed on lung tissues. Firstly, we detected the mRNA levels of ZEB1 and ZEB2 to evaluate the gene regulation activity of ZPM@PDE-siZEB1/2 in the lungs (Figure 7A). The mRNA levels of ZEB1 and ZEB2 were significantly downregulated in the LPS + ZPM@PDE-siZEB1/2 group compared to the control and LPS + Naked siZEB1/2 groups. Then, the immunohistochemical staining results showed that the lung tissues were dark brown stained in LPS + Naked siZEB1/2 mice compared to controls (Figure 7B), indicating that both ZEB1 and ZEB2 were highly expressed in these lungs. Meanwhile, the LPS + ZPM@PDE-siZEB1/2 group presented lighter brown staining, indicating decreased expression of ZEB1 and ZEB2. At last, the protein levels of ZEB1/2 (Figures 7C,D) were consist with the results of real-time qPCR and immunohistochemical staining. Collectively, the ZEB1 and ZEB2 expression levels could be effectively inhibited after being treated with ZPM@PDE-siZEB1/2, which verified the superb ZEB1 and ZEB2 silencing capacity and efficiency of the ZPM@PDE-siZEB1/2 as a new strategy for promising ALI treatment. After cellular entry, the entrapped siRNA/siRNA-carrier vesicles can be transported to early endosomes that mature into late endosomes (pH 4.0–6.5), then enter into lysosomes (pH ∼ 4.5) containing nucleases and other degradative enzymes (Zoulikha et al., 2022). Thus, ZPM@PDE-siZEB1/2 could enter cells via endocytosis and rapidly escape from endo/lysosomes via proton sponge effect of PDEA segments and osmolarity disruption effect of ZPM on the endosomal membrane. Then, the released siZEB1/2 could effectively knock down the level of ZEB1/2. ZEB1 and ZEB2 both are important regulators in the regulation of E-cadherin expression and EMT (Chilosi et al., 2017). When EMT occurs, the expression of E-cadherin was decreased, while the expression of ZEB1/2 was increased (Cao et al., 2018). EMT appeared in the early stage of pulmonary fibrosis caused by ALI, so silencing ZEB1/2 could alleviate early LPS-induced pulmonary fibrosis in ALI.

## Conclusion

In summary, we developed a novel NMOFs-based non-viral vector system to deliver siRNA for ALI treatment, which could protect the siRNA from enzymatic degradation in stable physiological pH 7.4 and decrease the cytotoxicity of bPEI. This system could effectively deliver siZEB1/2 into A549 cells for siRNA transfection and gene silencing. Besides, in ALI model mice, intratracheal administration of ZPM@PDE-siZEB1/2 could simultaneously deliver siZEB1 and siZEB2 into the inflammatory lung and effectively silence ZEB1/2, thereby delaying the progression of LPS-induced early pulmonary fibrosis in ALI mice. We did not only demonstrate that ZEB1/2 is a therapy target for ALI but also developed a promising siRNA delivery system for inflamed lung delivery to attenuate the progression of early pulmonary fibrosis during ALI. Finally, our delivery system might also have great potential in the treatment of COVID-19 infection-induced ALI/ARDS, inflammatory-related diseases, and tumors.

## Data Availability

The original contributions presented in the study are included in the article/Supplementary Material; further inquiries can be directed to the corresponding authors.
