# Supplementary material for: Nanoscale Porphyrin Metal-Organic Frameworks Deliver siRNA for Alleviating Early Pulmonary Fibrosis in Acute Lung Injury
Source: Front Bioeng Biotechnol. 2022 Jul 18;10:939312. doi: 10.3389/fbioe.2022.939312 (PMC9339993; doi:10.3389/fbioe.2022.939312)
Supplement: Supplementary file 1 [file DataSheet1.docx]

Supplementary Material


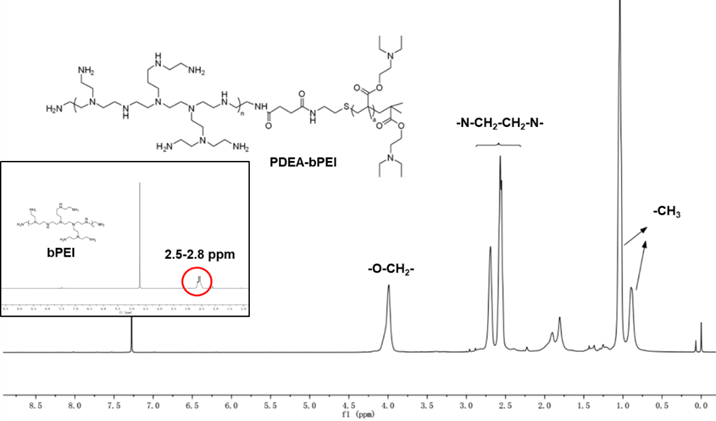


**Figure S**1. ^1^H-NMR spectra of PDEA-bPEI.


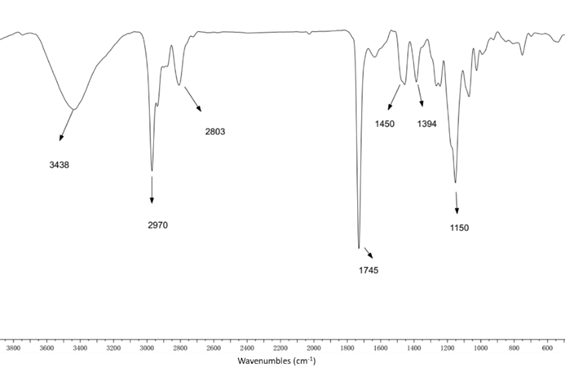


**Figure S2.** FT-IR spectra of PDEA-bPEI.

**Table S1.** Primers used in this study.

| **Gene name** | **Forward primer** | **Reverse primer** |
| --- | --- | --- |
| m-ZBE1 | CAATGATCAGCCTCAATCTGCA | TCATCATGACTGCTGGCTTC |
| m-ZBE2 | CGACACGGCCATTATTTACC | GGCAAAAGCATCTGGAGTTC |
| m-β-actin | CCTCTATGCCAACACAGT | AGCCACCAATCCACACAG |
| m-GAPDH | GGTGAAGGTCGGTGTGAACG | CTCGCTCCTGGAAGATGGTG |
| h-ZBE1 | AATGATCAGCCTCAATCTGCA | CCATTGGTGGTTGATCCCA |
| h-ZBE2 | AAGCCCCATCAACCCATACAAG | AAATTCCTGAGGAAGGCCCA |
| h-GAPDH | TCGACAGTCAGCCGCATCTTCTTT | GCCCAATACGACCAAATCCGTTGA |
| h-β-actin | CATGTACGTTGCTATCCAGGC | CTCCTTAATGTCACGCACGAT |

**Abbreviations:** m, mouse; h, human
